# Dynamic mitral regurgitation unmasked by handgrip exercise is linked with outcomes in (non-) dilated cardiomyopathy

**DOI:** 10.1016/j.ijcha.2025.101715

**Published:** 2025-06-02

**Authors:** Fabian Voß, Niklas Guenther, Lucas Christian, Elric Zweck, Jafer Haschemi, Christian Schulze, Ralf Westenfeld, Patrick Horn, Malte Kelm, Amin Polzin, Maximilian Spieker

**Affiliations:** a Division of Cardiology, Pulmonology and Vascular Medicine, University Hospital Duesseldorf, Heinrich-Heine University Duesseldorf, Medical Faculty, Moorenstraße 5, 40225 Duesseldorf, Germany; b Division of Cardiology, Vascular Medicine and Intensive Care Medicine, University Hospital Jena, Medical Faculty, Am Klinikum 1, 07747 Jena, Germany; c Cardiovascular Research Institute Duesseldorf, Medical Faculty, Heinrich-Heine University, Moorenstraße 5, 40225 Duesseldorf, Germany

## Abstract

**Background:**

Recent studies suggest that secondary mitral regurgitation (MR) is a dynamic condition influenced by global and regional left ventricular (LV) remodeling as well as by mitral valvular deformation. Exercise testing is crucial in assessing the hemodynamic relevance of MR and is recommended by current guidelines. However, data are still lacking on the prevalence and prognostic impact of dynamic MR in patients with non-ischemic cardiomyopathy.

**Aims:**

We aimed to assess the prevalence, hemodynamic consequences, and prognostic impact of exercise-induced changes in patients with MR and hypokinetic non-dilated and dilated cardiomyopathy.

**Methods:**

Patients with hypokinetic non-dilated and dilated cardiomyopathy and at least mild MR who underwent handgrip echocardiography at the University Hospital Duesseldorf between January 2018 and September 2021 were enrolled. Follow-up was performed at one year.

**Results:**

Fifty-eight patients were included (median age 73 [65;81] years; 41 % female; mean LVEF 37 ± 10 %). At rest, 28 patients (48 %) presented with mild MR and 30 patients with moderate MR (52 %). Fifteen patients (26 %) with non-severe MR at rest, developed severe MR during handgrip exercise. Patients with dynamic MR had larger left ventricular volumes, increased mitral annular diameter, and more advanced mitral valve tenting during exercise than those without dynamic MR. Patients with dynamic MR were more likely to undergo MV surgery/interventions (Chi^2^ 23.19; log-rank test p < 0.001).

**Conclusion:**

The hemodynamic changes provoked by isometric exercise unmasked dynamic MR in a significant number of patients without severe MR at rest. These data may have implications for therapeutic decision-making in symptomatic patients with non-severe MR at rest.

## 1 Introduction

Non-ischemic cardiomyopathy represents a complex and heterogeneous group of heart diseases characterized by structural and functional abnormalities of the myocardium, often leading to progressive heart failure. Dilated cardiomyopathy (DCM) and hypokinetic non-dilated cardiomyopathy (HNDCM) represent the most common etiologies. Among the various pathological mechanisms contributing to the deterioration of cardiac function in DCM and HNDCM, mitral regurgitation (MR) has emerged as a critical factor warranting clinical attention [1]. Conventionally, the assessment of MR severity has relied on resting echocardiography, which may not fully capture the dynamic and exercise-induced changes in MR contributing to patient reported symptoms and clinical outcomes. Previous studies have demonstrated the prognostic benefit of bicycle exercise testing in patients with primary and secondary MR [2,3]. Thus, current guidelines recommend exercise testing in various clinical scenarios in patients with valvular heart disease [4,5].

Isometric handgrip exercise represents an alternative exercise modality that can be performed fast without the need of an ergometer [6,7]. Until now, there are no data on the prognostic impact of dynamic MR in patients with DCM and HNDCM. We hypothesized that handgrip exercise during echocardiography may unmask dynamic MR in a significant proportion of patients, which could potentially alter therapeutic decision-making. In this regard, we aim to assess the prevalence, hemodynamic consequences and prognostic impact of dynamic MR in patients with DCM and HNDCM.

## 2 Methods

### 2.1 Study population

Consecutive patients undergoing exercise echocardiography at the university hospital Duesseldorf, Germany were enrolled between 2018 and 2021. In all patients, echocardiography at rest and during standardized handgrip exercise was performed. Exercise echocardiography was performed in heart failure patients with DCM and HNDCM, when there was a discrepancy between symptoms and echocardiographic findings at rest, rising the suspicion of a dynamic component. Only patients with reduced left ventricular ejection fraction (LVEF) < 50 %, and at least mild MR at rest were included. In patients with concomitant coronary artery disease significant coronary stenosis was excluded by coronary angiography. Patients with prior coronary intervention or myocardial infarction were excluded. The study was approved by the ethics committee of the Heinrich-Heine University Duesseldorf (No. 2018-117_1) and executed in accordance with the Declaration of Helsinki.

### 2.2 Echocardiographic examinations

Echocardiographic examinations were performed using a GE Vivid E90 (Chicago, Illinois, United States). All echocardiographic data were obtained in digital form and stored on a workstation for offline analysis (IntelliSpace Cardiovascular, Version 3.2, Philips). Assessment of MR was performed according to current ESC guidelines [5]. An integrative approach using semi-quantitative and at least one quantitative parameter was used to assess the severity of MR, which was graded mild, moderate or severe. Non-ischemic MR was defined in patients with known DCM and HNDCM with the mitral valve itself remaining intact (Carpentier class I). DCM and HNDCM were defined according to the recent ESC recommendations [8]. Mitral annulus was measured at end-diastole in the parasternal long-axis view and in apical four chamber view and then averaged. Mitral valve (MV) tenting was measured using established MV tenting parameters (tenting area; tenting height) and assessed in the parasternal long axis view during mid-systole according to the recommendations of the American Society of Echocardiography [9].

Left and right ventricular volumes and function were assessed according to current recommendations. Systolic pulmonary artery pressure (SPAP) was estimated from the regurgitant jet of tricuspid regurgitation (TR) with peak systolic *trans*-tricuspid pressure gradient (TTPG) calculated by the modified Bernoulli equation.

### 2.3 Isometric handgrip testing

Following echocardiographic examination at rest, handgrip exercise was conducted according to a standardized protocol with a handgrip dynamometer (Jamar® Hydraulic Hand Dynamometer, Sammons Preston Inc.). The patient lays on his left side during the whole examination and pushes the handgrip dynamometer with one hand. To assess maximal handgrip strength, the patient was asked to push the dynamometer with maximum effort. Then handgrip exercise was carried out at 30 % force for three to five minutes while the patient laid on his left side [10]. Echocardiographic images were obtained after two minutes of exercise until peak exercise. Blood pressure and heart rate were recorded at rest and during peak exercise. Medical therapy (including ß-blockers) remained unchanged for the exercise test.

### 2.4 Follow-up

The clinical course of patients was assessed by follow-up examinations and phone calls to the referring cardiologists, primary physicians, or the patients themselves. We assessed a composite of all-cause mortality, HF-associated hospitalizations, MV surgery, transcatheter edge-to-edge repair (TEER), left ventricular assist device implantation and heart transplantation during follow-up as the combined endpoint.

### 2.5 Statistical analysis

Percentages were reported to describe categorical variables and median with interquartile range or mean ± standard deviation was reported for continuous variables. Normality distribution of continuous variables was assessed with the Kolmogorov-Smirnov Test. Comparison between two groups were performed using the chi-square test (or Fisher’s exact test if the expected count was less than five per cell) for categorical variables. Differences in continuous variables between two groups were compared for significance with a two-tailed paired *t* test. Kaplan-Meier analysis was used to evaluate the event-free rate. Cox regression analysis was used to test for correlations between continuous variables and outcomes. For all analyses, p-values of < 0.05 were considered to be statistically significant. All analyses were performed using Sigma Plot (Version 11.0; Systat Software Ltd. Inpixon GmbH, Duesseldorf, Germany) and GraphPad Prism (Version 7; Graphpad Software, San Diego, California, USA).

## 3 Results

### 3.1 Study population

We screened 539 patients that underwent echocardiography at rest and during handgrip exercise. After excluding 94 patients (because of image quality (n = 50); handgrip exercise physically not feasible (n = 22); severe aortic stenosis (n = 2); severe mitral stenosis (n = 1), previous mitral valve repair (n = 19)) the study cohort included 445 patients. Fifty-eight out of these patients had DCM or HNDCM with at least mild MR and were included in final analysis. Patient characteristics are presented in Table 1. Mean age was 73 (IQR 65–81) years, 41 % were female. Mean LVEF was 37 ± 9 %. In this regard, 43 patients (74 %) presented with HFmrEF, and 15 patients (26 %) presented with HFrEF. Twenty-six patients (45 %) had HNDCM and 32 patients (55 %) were diagnosed with DCM.

**Table 1:** Baseline patient characteristics.

Demographics	All Patients N = 58 (100.0)
Sex female, n (%)	24 (41.4)
BMI (kg/m^2^)	26.5 (23.1–29.4)
Age (years)	73 (65–81)
Diabetes Mellitus, n (%)	12 (20.1)
Hypertension, n (%)	34 (58.6)
Smoking, n (%)	12 (20.1)
DCM, n (%)	32 (55.2 %)
HNDCM, n (%)	26 (44.8 %)
CAD, n (%)	25 (43.0)
History of MI, n (%)	0 (0.0)
History of PCI, n (%)	0 (0.0)
History of CABG, n (%)	0 (0.0)
History of VS, n (%)	2 (3.5)
Pacemaker, n (%)	4 (6.9)
ICD, n (%)	12 (20.7)
CRT, n (%)	11 (19.0)
NYHA 1, n (%)	5 (8.6)
NYHA 2, n (%)	17 (29.3)
NYHA 3, n (%)	36 (62.1)
Atrial Fibrillation, n (%)	31 (53.5)
Medication	
ß-Blocker, n (%)	52 (89.7)
ACE-I/AT-1 Blocker, n (%)	33 (56.9)
Aldosteron Antag., n (%)	33 (56.9)
Sacubitril/Valsartan, n (%)	18 (31.0)
Diuretic, n (%)	59 (84.5)
Laboratory Markers	
Serum Creatinine (mg/dl)	1.3 (1.0–1.5)
eGFR (mg/dl/1.73 m^2^)	53 (39–74)
Hemoglobin (mg/dl)	13.2 (11.7–14.8)
NT-proBNP (ng/ml)	4347 (1590–8549)

Abbreviations: BMI = body mass index; CAD = coronary artery disease; MI = myocardial infarction; PCI = percutaneous coronary intervention; CAGB = coronary artery bypass grafting; VS = valve surgery; ICD = internal cardiac defibrillator; CRT = cardiac resynchronization therapy; eGFR = estimated glomerular filtration rate; NT-proBNP=N-terminal pro brain natriuretic peptide.

### 3.2 Echocardiographic parameters at rest and during exercise

Twenty-eight patients (48 %) presented with mild MR, and 30 patients (52 %) had moderate MR (41 %) at rest. Detailed data of mitral valve parameters were shown in Table 2. Forty-three percent of patients presented with concomitant moderate to severe TR. Mean SPAP was 41 ± 10 mmHg.

**Table 2:** Echocardiographic parameters at rest and during handgrip exercise.

Variable	Rest	Exercise	p-Value
Heart Rate (bpm)	78 ± 14	95 ± 24	**<0.001**
Systolic BP (mmHg)	113 ± 23	127 ± 24	**<0.001**
Diastolic BP (mmHg)	67 ± 17	77 ± 19	**0.002**
RPP (mmHg*bpm)	8766 ± 2311	12040 ± 3930	**<0.001**
LAVi (ml/m^2^)	51 ± 18	52 ± 21	0.899
LVEDVi (ml/m^2^)	90 ± 38	92 ± 41	0.788
LVESVi (ml/m^2^)	59 ± 35	61 ± 32	0.565
LVSVi (ml/m^2^)	30 ± 12	30 ± 13	0.762
LVEF (%)	37 ± 9	36 ± 12	0.222
LV Sphericity Index	1.42 ± 0.23	1.49 ± 0.19	0.059
LV Forward Flow Index (ml/m^2^)	31 ± 12	28 ± 10	0.302
Cardiac Index (l/min/m^2^)	2.3 ± 1.0	2.8 ± 1.4	**0.048**
SVR (dyn*sec*cm^−2^)	1530 ± 648	1450 ± 526	0.690
RAVi (ml/m^2^)	42 ± 19	42 ± 19	0.783
RVEDDi (mm/m^2^)	22 ± 4	22 ± 4	0.243
TAPSE (mm)	19 ± 6	18 ± 6	0.262
FAC (%)	34 ± 10	35 ± 12	0.972
SPAP (mmHg)	41 ± 10	48 ± 12	**<0.001**
Mitral Regurgitation			**<0.001**
Mild, n (%)	28 (48.3)	15 (32.8)	
Moderate, n (%)	30 (51.7)	24 (41.1)	
Severe, n (%)	0	19 (25.9)	
Vena Contracta (mm)	5.2 ± 1.4	6.0 ± 1.7	**0.017**
MR EROA (mm^2^)	16.0 ± 6.0	21.1 ± 8.8	**<0.001**
MR Vol (ml)	25 ± 9	33 ± 13	**<0.001**
Annulus Diameter (mm)	39.3 ± 4.3	40.0 ± 4.5	0.372
Tenting Height (mm)	9.5 ± 2.3	10.3 ± 3.2	**0.034**
Tenting Area (cm^2^)	2.5 ± 0.9	2.8 ± 1.0	**0.045**
Tricuspid Regurgitation			0.603
No TR, n (%)	19	10.5	
Mild, n (%)	37.9	42.1	
Moderate, n (%)	31	36.8	
Severe, n (%)	12.1	10.5	

Abbreviations: BP = blood pressure; RPP = rate pressure product; LAVi = left atrial volume index; LVEDVi = left ventricular end-diastolic volume index; LVESVi = left ventricular end-systolic volume index; LVEF = left ventricular ejection fraction; LVSVi = left ventricular stroke volume index; RAVi = right atrial volume index; RVEDDi = right ventricular end-diastolic diameter index; TAPSE = tricuspid annular plane systolic excursion; FAC = fractional area change; SPAP = systolic pulmonary artery pressure; EROA = effective regurgitant orifice area; Vol = volume; IPM Distance = interpapillary muscle distance; TR = tricuspid regurgitation.

**Fig. 1:**
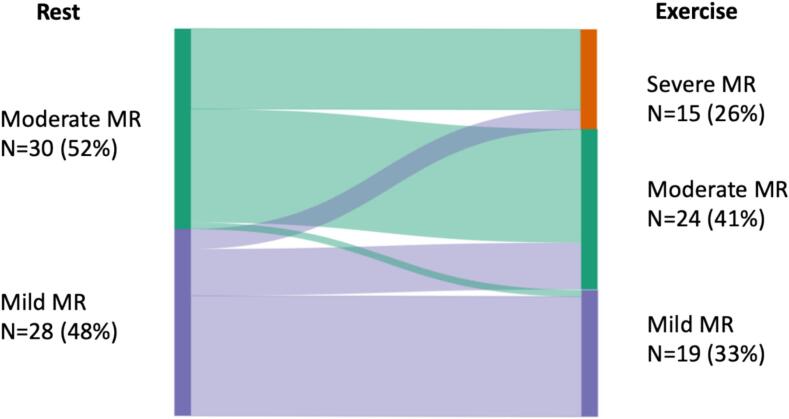
Distribution of MR severity at rest and during handgrip exercise. Abbreviations: MR = mitral regurgitation.

Isometric handgrip exercise led to a meaningful hemodynamic response: heart rate, systolic and diastolic blood pressure, and rate pressure product increased (all p < 0.05, Table 2). This was accompanied by a re-classification of MR severity in 22 patients (38 %) (Fig. 1). Fifteen patients (26 %) with non-severe MR at rest, developed dynamic severe MR during exercise (Fig. 1). Of these patients, six patients (40 %) had HNDCM and nine patients (60 %) had DCM. In the overall cohort, there was an increase in cardiac index (+0.5 ± 1.3 l/min/m^2^; p = 0.048) that was caused by an increase in heart rate (+17 ± 20/min; p < 0.001), and not by LV forward flow (−2 ± 12 ml/m^2^; p = 0.302), while RV volumes and function did not change. SPAP increased by 7 ± 12 mmHg from rest to exercise. Detailed changes of echocardiographic parameters from rest to exercise were shown in Table 2.

### 3.3 Mechanisms and hemodynamic consequences of dynamic MR

Patients with dynamic severe MR had larger mitral annulus diameter at rest, while LA volume (LAVi) also tended to be larger (Table 3). In line with this, LV volumes (LVEDVi and LVESVi) at rest were numerically larger in patients with dynamic severe MR compared to those with non-severe MR (Table 3). There was no difference in pulmonary pressures (SPAP), right heart dimensions and function between the groups (Table 3).

**Table 3:** Echocardiographic parameters at rest according to MR severity.

Variable	Non-severe MR (N = 43)	Dynamic Severe MR (N = 15)	p-Value
Heart Rate (bpm)	79 ± 15	75.2 ± 11.1	0.37
Systolic BP (mmHg)	112 ± 24.2	117 ± 20.5	0.47
Diastolic BP (mmHg)	66.3 ± 13.7	70.4 ± 23.3	0.42
RPP (mmHg*min^−1^)	8763 ± 2432	8775 ± 2010	0.99
LAVi (ml/m^2^)	48.5 ± 16.8	57.9 ± 20.4	0.13
LVEDVi (ml/m^2^)	86.8 ± 39.1	107 ± 42.8	0.08
LVESVi (ml/m^2^)	54.6 ± 33.6	72.3 ± 34.8	0.07
LVSVi (ml/m^2^)	29.6 ± 12.6	31 ± 10.3	0.71
LVEF (%)	37.6 ± 10	25.9 ± 9.6	0.56
Forward LVSVi ((ml/m^2^)	30.4 ± 11.8	31.5 ± 14	0.91
Cardiac Index (l/min/m^2^)	2.4 ± 1	2.4 ± 1.1	0.97
SVR (dyn*sec*cm^−5^)	1576 ± 704	1400 ± 450	0.62
RAVi (ml/m^2^)	40.1 ± 20	45.8 ± 17.6	0.34
RVEDDi (mm/m^2^)	21.4 ± 4.2	23 ± 6	0.27
TAPSE (mm)	18.6 ± 6.3	18.3 ± 4.9	0.9
FAC (%)	34.9 ± 10.2	31.2 ± 10.6	0.66
SPAP (mmHg)	40.6 ± 9.7	43.3 ± 10.3	0.39
Mitral Regurgitation			**0.015**
Mild, n (%)	26 (59.1)	3 (20.0)	
Moderate, n (%)	17 (40.9)	12 (80.0)	
Severe, n (%)	0	0	
MR Vena Contracta (mm)	4.9 ± 1.4	6.2 ± 1	**0.004**
MR EROA (mm^2^)	0.14 ± 0.05	0.21 ± 0.06	**< 0.001**
MR Vol (ml)	21.9 ± 6.97	34.3 ± 7.39	**< 0.001**
Annulus Diameter (mm)	38.5 ± 4.4	41.6 ± 3.3	**0.01**
AML Length (mm)	29.5 ± 5.6	30.3 ± 4.1	0.64
PML Length (mm)	17.2 ± 4.8	17.7 ± 2.9	0.7
Leaflet-to-Annulus Index	1.21 ± 0.21	1.16 ± 0.19	0.45
Tenting Height (mm)	9.3 ± 2.5	10.1 ± 1.3	0.23
Tenting Area (cm^2^)	2.38 ± 0.9	2.85 ± 0.8	0.1
TR Grade (n)			0.56
No TR, n (%)	23.3	6.7	
Mild, n (%)	34.9	46.7	
Moderate, n (%)	30.2	33.3	
Severe, n (%)	11.6	13.3	

Abbreviations: Please see Table 2.

During exercise, LVEDVi (113 ± 51 ml/m^2^ vs. 81 ± 28 ml/m^2^; p = 0.005) and LVESVi (80 ± 28 ml/m^2^ vs. 54 ± 24 ml/m^2^; p = 0.006) were increased in patients with dynamic severe MR compared to those with non-severe MR. Similarly, mitral annulus diameter (42 ± 3.7 mm vs. 39.3 ± 4.6 mm; p = 0.076) as well as tenting area (3.3 ± 0.9 cm^2^ vs. 2.5 ± 0.9 cm^2^; p = 0.052) and tenting height (12.5 ± 2.9 mm vs. 9.2 ± 2.8 mm; p = 0.005) were larger in patients with dynamic severe MR.

**Fig. 2:**
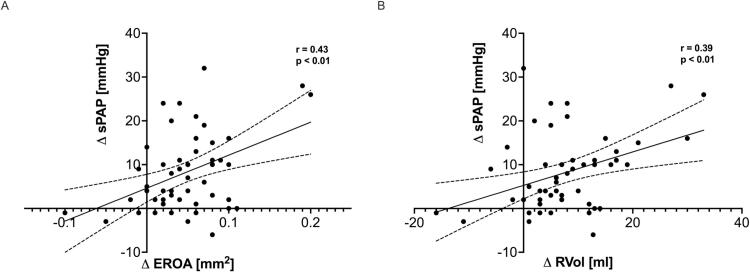
Association between changes from rest to exercise in MR severity (assessed by (A) EROA and (B) RVol) and pulmonary pressures (SPAP). Abbreviations: SPAP = systolic pulmonary artery pressure; EROA = effective regurgitant orifice area; RVol = regurgitation volume.

There was no difference in LV forward flow and cardiac index between the groups during exercise. Patients with dynamic severe MR showed increased exercise SPAP compared to those without, while RV dimensions and function were similar between both groups. Furthermore, the increase in MR severity (by effective regurgitation orifice area (EROA) and regurgitation volume (RVol)) correlated with the change in SPAP (Fig. 2).

### 3.4 Clinical outcomes according to the presence of dynamic MR

**Fig. 3:**
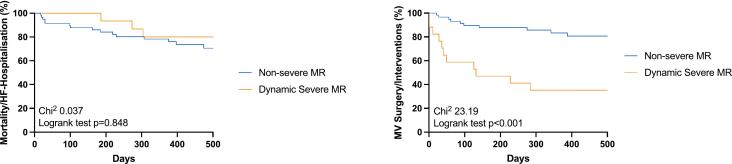
Kaplan-Meier survival analysis regarding combined events (**A**), and MV surgery/interventions (**B**) in patients with and without dynamic severe MR. Abbreviations: MR = mitral regurgitation; MV = mitral valve.

One-year follow-up (median 442 (334–691) days) was completed in all patients (100 %). Thirty-eight patients (66 %) experienced the combined endpoint: three patients died (5 %), 14 patients (24 %) were re-admitted to hospital due to heart failure symptoms, 17 patients (29 %) underwent MV TEER, one patient (2 %) received MV surgery, two patients (3 %) underwent LVAD implantation, and one patient (2 %) underwent heart transplantation. There was no difference in the combined endpoint of all-cause mortality and heart-failure hospitalizations regarding patients with non-severe MR and patients with dynamic severe MR (Chi^2^ 0.037; log-rank test p = 0.85) (Fig. 3A). As expected, patients with dynamic severe MR more often underwent MV surgery/interventions compared to patients with non-severe MR (Chi^2^ 23.19; log-rank test p < 0.001) (Fig. 3B). Cox regression analysis was used to analyze the relationship between the change in EROA/RVol during exercise and clinical outcomes. The change in EROA/RVol was correlated with MV surgery/ interventions (ΔEROA: HR 1.145 (1.051–1.387), p < 0.001; ΔRVol: HR 1.098 (1.045–1.151), p < 0.001), but not with mortality and heart failure hospitalizations (ΔEROA: HR 1.022 (0.928–1.105), p = 0.631; ΔRVol: HR 1.016 (0.955–1.079), p = 0.612). As explorative analyses, the incidence of the combined endpoint was calculated and was less frequently observed in patients who underwent MV surgery/TEER compared to those who did not: three out of 18 patients (16.7 %) who underwent MV surgery/TEER experienced an adverse event compared to 11 out of 38 patients (29.0 %) of those patients who did not receive MV surgery/TEER (Chi^2^ 1.167; log-rank test p = 0.280).

## 4 Discussion

In this study, we evaluated the role of exercise echocardiography for the assessment of dynamic MR in patients with DCM and HNDCM. We demonstrated 1.) that handgrip exercise unmasks dynamic severe MR in one out of four patients with non-severe MR at rest; 2.) that dynamic severe MR was associated with global and regional adverse LV remodeling during exercise; and 3.) that dynamic severe MR was associated with increased rates of MV surgery/interventions during follow-up compared to those with non-severe MR.

### 4.1 Prevalence of dynamic MR

Until now, studies investigating the role of exercise testing in patients with DCM and HNDCM for the assessment of dynamic MR remain scarce. D'Andrea et al. reported an increase in MR severity in 78 % of patients focusing on bicycle exercise testing [11]. Yamano et al. demonstrated an increase in MR severity in 23 of 32 patients with DCM. This is the first study yet, investigating the role of dynamic MR in patients with DCM and HNDCM using isometric handgrip exercise [12]. Here, we could demonstrate that 26 % of patients with non-severe MR at rest developed dynamic severe MR during exercise. Furthermore, in our study handgrip testing led to a re-classification of MR severity in 38 % of patients. Our results highlight the benefit of the addition of exercise echocardiography to echocardiography at rest in patients with DCM and HNDCM. Symptomatic patients with dynamic severe MR may benefit from intensified HF medication or early interventional strategies [13].

### 4.2 Mechanisms and hemodynamic consequences of dynamic MR

Predictors of exercise-induced increases in MR severity unmasked by *dynamic* bicycle exercise have been studied before [11,12]: an increase in EROA was associated with the enlargement of tenting area. Furthermore, the increase in LV dyssynchrony during exercise correlated with the increase in MR severity during exercise. This is the first study investigating the pathophysiological mechanisms of changes in MR provoked by *isometric* handgrip exercise in patients with DCM and HNDCM. While *dynamic* bicycle exercise leads to a decrease in afterload, an increase in contractility and venous return (increase in preload), that together may affect an increase in stroke volume, *isometric* handgrip exercise predominantly causes an increase in afterload. These different loading conditions may translate into different LV geometric changes during exercise, and precipitate exercise-induced changes in MR severity [6]. In the current study, mitral annular dimensions and LV volumes were associated with dynamic severe MR during exercise. Moreover, the change in MR severity was directly associated with the change in tenting area. Thus, both, regional and global LV remodeling seem to be responsible for worsening of MR during exercise. Future studies comparing both exercise modalities are necessary to further elucidate these findings. Isometric handgrip exercise causes an increased backward transmission and leads to a pressure load on the pulmonary circulation, that was more advanced in patients with dynamic severe MR (SPAP + 13 ± 10 mmHg) compared to those with non-severe MR (SPAP + 6 ± 8 mmHg) (Table 4). Moreover, there was a correlation between the change in MR severity from rest to exercise and the change in pulmonary pressures (Fig. 2). This may explain symptoms in patients with a discrepancy between symptoms and echocardiographic findings at rest.

**Table 4:** Exercise changes in echocardiographic parameters according to MR severity.

Variable	Non-severe MR (N = 43)	Dynamic Severe MR (N = 15)	p-Value
ΔHeart Rate (bpm)	16.4 ± 22.5	17.1 ± 11.9	0.43
Δ Systolic BP (mmHg)	12.9 ± 13.5	15.5 ± 10.1	0.38
Δ Diastolic BP (mmHg)	8.8 ± 14.8	8.7 ± 7.9	0.6
Δ RPP (mmHg*min^−1^)	3177 ± 3168	3407 ± 2320	0.51
Δ LAVi (ml/m^2^)	0.65 ± 16.8	0.47 ± 11.6	0.97
Δ LVEDVi (ml/m^2^)	−2.35 ± 19.4	5.59 ± 25.8	0.58
Δ LVESVi (ml/m^2^)	2.46 ± 17.3	7.44 ± 19.3	0.99
Δ LVSVi (ml/m^2^)	−1.94 ± 11.7	3.48 ± 18.9	0.21
Δ LVEF (%)	−1.17 ± 11	− 3.35 ± 9.1	0.52
Δ Forward LVSVi ((ml/m^2^)	−4.97 ± 7.76	−1.2 ± 9.75	0.24
Δ Cardiac Index (l/min/m^2^)	0.29 ± 1.2	0.37 ± 0.62	0.46
Δ SVR (dyn*sec*cm^−5^)	140 ± 522	−117 ± 337	0.2
Δ RAVi (ml/m^2^)	1.2 ± 14.3	−3.7 ± 9.5	0.22
Δ RVEDDi (mm/m^2^)	0.49 ± 3	0.61 ± 5.8	0.38
Δ TAPSE (mm)	−0.22 ± 4.47	0.36 ± 3.54	0.67
Δ FAC (%)	0.46 ± 10.7	2 ± 8.85	0.7
Δ SPAP (mmHg)	6.3 ± 7.6	12.9 ± 10.1	**0.02**
ΔΔ MR Vena Contracta (mm)	0.69 ± 1.33	0.9 ± 1.47	0.66
Δ MR EROA (mm^2^)	0.038 ± 0.05	0.087 ± 0.048	**<0.001**
Δ MR Vol (ml)	5.55 ± 7.04	14.5 ± 10.2	**<0.001**
Δ Annulus Diameter (mm)	0.8 ± 2.76	0.09 ± 3.57	0.98
Δ Tenting Height (mm)	−0.06 ± 1.83	2.35 ± 2.1	**0.009**
Δ Tenting Area (cm^2^)	0.14 ± 0.59	0.51 ± 0.73	0.16

Abbreviations: Please see Table 2.

### 4.3 Impact of exercise echocardiography on outcomes

In our cohort, there was no difference in clinical outcomes regarding the combined endpoint in patients with non-severe MR and patients with dynamic severe MR. However, exercise echocardiography unmasks a substantial proportion of patients (26 %) with non-severe MR at rest and dynamic severe MR during exercise. The recently published RESHAPE HF II trial showed improved outcomes after TEER in moderate to severe MR. [14] In contrast, former trials suggested only patients with severe (disproportionate) MR to benefit from MV interventions [15,16]. Dynamic MR maybe a partial explanation to these results, and therefore, exercise testing, may help to clarify this controversial issue in future randomized trials. As expected, MV surgery/intervention occurred more frequently during follow-up in patients with dynamic severe MR compared to the other group (Fig. 3b). Importantly, surgeons and interventionalists were not blinded to the results of exercise testing. Nevertheless, all patients were discussed within the local heart team and, therefore, all aspects of echocardiographic results, hemodynamics and patients’ symptoms were considered. Interestingly, patients who underwent MV surgery/intervention exhibited improved clinical outcomes compared to patients who did not. Although, randomized trials are needed to confirm this assumption, these results are in line with previous findings from Li et al. who investigated 112 patients with non-ischemic cardiomyopathy and significant MR [17]. They found that MV surgery/interventions were associated with better survival rates and lower HF hospitalizations compared to patients that were treated conservatively. In another study by Takeda et al., MV surgery led to LV reverse remodeling and the extent of reverse remodeling was related to mid-term mortality [18]. Together, there are only a few data on the prognostic importance of MV surgery/interventions in patients with DCM and HNDCM. This is the first study that demonstrated an incremental diagnostic value of isometric handgrip testing over echocardiography at rest in patients with DCM and HNDCM. However, since outcomes of patients with and without dynamic severe MR did not differ in our cohort, the prognostic impact of dynamic severe MR cannot be proven from this analysis alone, also the prognostic impact may have been diminished by early interventions among symptomatic patients. Further studies are warranted that randomize patients to additional exercise testing to further elucidate the role of exercise echocardiography in this patient cohort, before a widespread use of exercise testing can be recommended.

### 4.4 Limitations

Main limitation of the current study is that patients were not randomized to exercise testing. Thus, there might be a selection bias for patients undergoing MV surgery/interventions. In addition, exercise echocardiography was performed in patients with suspicion of a dynamic component that may have led to a selection bias since there was concern for dynamic MR that led to the exercise test being ordered and inclusion in the study. Cardiologists and cardiac surgeons were not blinded to the results of the exercise tests, thus, the decision for surgery/intervention might be by part influenced by the results of exercise echocardiography. Whether MV surgery/intervention among these patients has impact on patients’ outcomes in this specific cohort needs further investigation in randomized trials. That is why analyses of the combined endpoint after MV surgery/intervention can only be seen explorative and hypothesis-generating. Due to the relatively small sample size in our cohort, the prognostic value of isolated dynamic severe MR needs further validation e.g. in randomized trials or at least sensitivity analyses, which are of questionable value in this context since multiple – in part unknown factors – may influence patients‘ outcomes in this cohort.

## 5 Conclusions

Our results demonstrate that exercise echocardiography unmasks dynamic severe MR in a substantial proportion of DCM and HNDCM patients with non-severe MR at rest. MR severity during exercise was associated with MV surgery/interventions during follow-up. In addition, our results suggest the hypothesis that MV surgery/interventions are associated with improved clinical outcomes in patients with dynamic severe MR compared to those who are treated conservatively, but randomized clinical trials are needed to confirm this hypothesis. Thus, handgrip exercise might be a useful tool in symptomatic patients with suspicion of dynamic severe MR to help guiding further therapeutic decision making.

## CRediT authorship contribution statement

**Fabian Voß:** Writing – review & editing, Writing – original draft, Visualization, Validation, Supervision, Software, Resources, Project administration, Methodology, Investigation, Formal analysis, Data curation, Conceptualization. **Niklas Guenther:** Writing – review & editing, Visualization, Validation, Methodology, Formal analysis, Data curation, Conceptualization. **Lucas Christian:** Writing – review & editing, Visualization, Methodology, Formal analysis, Data curation. **Elric Zweck:** Writing – review & editing, Visualization, Validation, Supervision, Conceptualization. **Jafer Haschemi:** Writing – review & editing, Supervision, Resources, Project administration, Investigation, Formal analysis, Conceptualization. **Christian Schulze:** Writing – review & editing, Writing – original draft, Visualization, Validation, Supervision, Conceptualization. **Ralf Westenfeld:** Writing – review & editing, Writing – original draft, Validation, Supervision, Project administration, Investigation, Formal analysis, Conceptualization. **Patrick Horn:** Writing – review & editing, Visualization, Validation, Supervision, Resources, Methodology, Investigation, Conceptualization. **Malte Kelm:** Writing – review & editing, Writing – original draft, Visualization, Validation, Funding acquisition, Conceptualization. **Amin Polzin:** Writing – review & editing, Writing – original draft, Validation, Supervision, Project administration, Methodology, Funding acquisition, Data curation, Conceptualization. **Maximilian Spieker:** Writing – review & editing, Writing – original draft, Visualization, Validation, Supervision, Software, Resources, Project administration, Methodology, Investigation, Funding acquisition, Formal analysis, Data curation, Conceptualization.

## Declaration of competing interest

The authors declare that they have no known competing financial interests or personal relationships that could have appeared to influence the work reported in this paper.
